# Decision to delivery interval and associated factors for emergency cesarean section: a cross-sectional study

**DOI:** 10.1186/s12884-021-03706-8

**Published:** 2021-03-20

**Authors:** Tebabere Moltot Kitaw, Simachew Kassa Limenh, Fantahun Alemnew Chekole, Simegnew Asmer Getie, Belete Negese Gemeda, Abayneh Shewangzaw Engda

**Affiliations:** 1 Department of Midwifery, College of Health Science, Debre Berhan University, Debre Berhan, Ethiopia; 2 School of Health Sciences, College of medicine and health science, Bahir Dar University, Bahir Dar, Ethiopia; 3 Department of Nursing, College of Health Science, Debre Berhan University, Debre Berhan, Ethiopia

**Keywords:** Decision to delivery interval, Emergency cesarean section, Public hospitals, Bahir Dar, Ethiopia

## Abstract

**Background:**

Emergency cesarean section is a commonly performed surgical procedure in pregnant women with life-threatening conditions of the mother and/or fetus**.** According to the Royal College of Obstetricians and Gynecologists and the American College of Obstetricians and Gynecologists, decision to delivery interval for emergency cesarean sections should be within 30 min. It is an indicator of quality of care in maternity service, and if prolonged, it constitutes a third-degree delay. This study aimed to assess the decision to delivery interval and associated factors for emergency cesarean section in Bahir Dar City Public Hospitals, Ethiopia.

**Method:**

An institution-based cross-sectional study was conducted at Bahir Dar City Public Hospitals from February to May 2020. Study participants were selected using a systematic random sampling technique. A combination of observations and interviews was used to collect the data. Data entry and analysis were performed using Epi-data version 3.1 and SPSS version 25, respectively. Statistical significance was set at *p* < 0.05.

**Result:**

Decision-to-delivery interval below 30 min was observed in 20.3% [95% CI = 15.90–24.70%] of emergency cesarean section. The results showed that referral status [AOR = 2.5, 95% CI = 1.26–5.00], time of day of emergency cesarean section [AOR = 2.5, 95%CI = 1.26–4.92], status of surgeons [AOR = 2.95, 95%CI = 1.30–6.70], type of anesthesia [AOR = 4, 95% CI = 1.60–10.00] and transfer time [AOR = 5.26, 95% CI = 2.65–10.46] were factors significantly associated with the decision to delivery interval.

**Conclusion:**

Decision-to-delivery intervals were not achieved within the recommended time interval. Therefore, to address institutional delays in emergency cesarean section, providers and facilities should be better prepared in advance and ready for rapid emergency action.

**Supplementary Information:**

The online version contains supplementary material available at 10.1186/s12884-021-03706-8.

## Background

Emergency cesarean section (EmCS) is a surgical procedure that is performed when there is an immediate threat to the life of a fetus and/or woman [1]. The period between a decision to perform EmCS and the actual delivery of the neonate is called decision to delivery interval (DDI) [1, 2]. It includes patient and theater preparation time, anesthetic time, and the skin incision to delivery interval [3]. According to Royal College of Obstetricians and Gynecologists (RCOG) and American College of Obstetricians and Gynecologists (ACOG), the recommended decision to delivery interval (DDI) is within 30 min [1, 4].

Cesarean section (CS) can improve infant and/or maternal outcomes only when used appropriately [5]. Therefore, hospitals providing obstetric care should be able of respond to obstetric emergencies within the recommended time [2, 6, 7]. Despite this, in developing nations, reviews reported that difficulty in achieving the recommended 30 min DDI [8–11] and fetal deaths occurred while waiting for EmCS [9]. This indicates that in developing nations, women in labor need timely access to skilled care.

In Ethiopia, the EmCS access is high [12, 13]. However, similar to other developing nations, poor neonatal outcomes after delivery by EmCS are high [14]. This results in psychological and physical trauma to the mother. Even though the procedure of cesarean section is complex and multidisciplinary, and some laboring women need stabilization before the procedure [3, 9]. In case of an emergency cesarean section, DDI must be considered to be completed in the target [4]. DDI remains an important indicator for evaluating the quality of maternity care in EmCS [1, 14, 15]. Therefore, this study aimed to assess DDI and its associated factors in Bahir Dar City Public Hospitals, Ethiopia.

## Methods

### Study setting, period, and participants

An institution-based cross-sectional study was conducted from February 26, 2020, to May 26, 2020, in Bahir Dar city public hospitals, the capital of Amhara region, northwest Ethiopia. This city has three public hospitals; among these two are referral hospitals and one primary hospital. These hospitals service for over 7,000,000 populations and have a total of 160 skilled birth attendants. All these hospitals have 52 beds for labor and delivery services and approximately 13, 920 deliveries per year. All women who underwent EmCS at Bahir Dar City Public Hospitals were the source population. Therefore, all women who underwent EmCS during the study period at Bahir Dar City public hospitals were the study population. In this study, women who underwent EmCS during the study period were included.

### Sample size and sampling procedure

The sample size was calculated using the single population proportion formula with the assumption of 95% CI, and 12% of women who had recommended decision to delivery interval in Tanzania [10]. A 10% non-response rate and 3.7% margin of error were used to obtain a sample size of 327. The total sample size was proportionally allocated for all three hospitals based on the last year’s similar three-month EmCS report. Eligible women in each hospital were invited to participate using a systematic random sampling technique. The sampling fraction was determined by dividing the total three-month EmCS rate in each hospital by the sample size, which was proportionally allocated in each hospital. The first participant was selected using the lottery method and every 2nd interval in each hospital was included in the study.

### Data collection tools and technique

The questionnaire was developed after an extensive review of the literature (Supplementary file 1). The tool was modified and finalized based on the suggestions and recommendations of the local experts. A structured interview questionnaire and observational checklist were used for data collection. One supervisor and two Bachelor of Science (BSc) midwives were employed as data collectors. Training was provided to the data collectors and the research supervisor. A pretest was conducted with 33(10%) women who underwent emergency cesarean section randomly chosen from a population outside the study area. Questionnaires were cleaned daily by the data collection supervisor under the primary investigator’s oversight. Questionnaires were checked for completeness and consistency, and when missing items were discovered, the items were collected and coded appropriately. The collected data were checked for completeness and consistency by the supervisor under the guidance of the primary investigator.

### Variables and measurements

Independent variables in this study were classified into seven sections: socio-demographic factors, including age, educational status, marital status, place of residence, and occupation. Obstetric factors included ANC follow-up, gravidity, parity, and number of live children. Presence of cesarean scare, stage of labor at decision, referral status, and an indication of EmCS. The Time of day and day of the week of EmCS, preoperative related factors include preoperative stabilization need, hesitation for consent, transfer time interval, and presence of material for preparation. Surgeon status, type of anesthesia, and operative room-related variables were also examined as potential predictors to assess the decision to delivery interval. The dependent variable in this study was decision to delivery interval.

### Operational definition

#### Emergency cesarean section

This is based on a binary classification system for cesarean section [6].

#### DDI

After calculating to the nearest minute, 30 min was used as a cutoff point to say recommended/delayed [4, 16].

#### Transfer time

The time taken from the decision for EmCS to arrival in the operation theater and 15 min was used as the cut-off point to say delayed or not [9, 17].

#### Operation time

Time taken from skin incision to delivery of the fetus and 5 min used as a cutoff point to say delayed or not [18].

### Data management and analysis

Before analysis, completeness and consistency of each questioner were checked, coded and entered into Epi data version 3.1, and then exported to SPSS version 25 for data cleaning, recoding and analysis. Categorical variables are presented as frequencies and percentages. Bivariable and multivariable logistic regression analysis were performed to identify the independent predictors of the outcome variable. Hosmer and Lemeshow goodness of fit test (*p* = 0.85) and variance inflation factor were done to check model fitness and problem of multicollinearity respectively. Variables with a *p*-value ≤0.25, in the bivariable logistic regression analysis, were entered into a multivariable logistic regression model. Variables with a p-value < 0.05 with 95% confidence interval (CI) for Adjusted odds ratio (AOR) were considered statistically significant.

## Result

### Socio-demographic characteristics

A total of three hundred twenty-five women were enrolled in this study, with a response rate of 99.4%. The median age (interquartile range (IQR)) of the respondents was 26 (23–30) years. Nearly one-third (37.8%) of the respondents were between the ages of 25–29 years and more than two-thirds (70.2%) of the study subjects were urban residents (Table 1).

**Table 1:** Socio-demographic characteristics of respondents (*n* = 325)

Variable	Categories	n (%)
AGE in Years	< 20 (all are above 18)	13(4)
	20–24	94(28.9)
	25–29	123(37.8)
	30–34	61(18.8)
	≥35	34(10.5)
Marital Status	Single	14(4.3)
	Married	304(93.5)
	Divorced	7(2.2)
Educational Status	No Formal Education	111(34.2)
	Primary School	70(21.5)
	Secondary School	69(21.2)
	Collage / university	75(23.1)
Occupational Status	Government Employee	53(16.3)
	House Wife	192(59.1)
	Daily Labor	30(9.2)
	Merchant	39(12)
	Others^a^	11(3.4)
Place of Residence	Urban	228(70.2)
	Rural	97(29.8)

^a^(student, non-governmental employee)

### Obstetrical characteristics of respondents

The findings showed that 48.3% of the respondents were nulliparous. Almost two-thirds of the respondents had four or more antenatal care (ANC) visits. Thirty-nine (12%) participants had a previous history of cesarean section (Table 2).

**Table 2:** Obstetrical characteristics of respondents (*n* = 325)

Characteristics	Categories	n(%)
Gravidity	Primigravidia	150(46.2)
	Multigravida	150(46.2)
	Grand multipara	25(7.6)
Parity	Nulliparous	157(48.3)
	Primiparous	80(24.6)
	Multiparous	73(22.5)
	Grand multiparous	15(4.6)
ANC follow up	First visit	6(1.8)
	Second visit	16(4.9)
	Third visit	64(19.7)
	Fourth and above	222(68.3)
	No ANC follow up	17(5.3)
BPCR^a^(*n* = 308)	Yes	233(75.65)
	No	75(24.35)
Scared Uterus	Yes	39(12%)
	No	286(88%)

^a^*BPCR* Birth preparedness and complication readiness plan

In this study, non-reassuring fetal heartbeat patterns (NRFHBP) were the most common indication for emergency cesarean section, which accounted for 78(24%) of cases followed by cephalopelvic disproportion (CPD) 64(19.7%). The lowest median time was recorded for cord prolapse, whereas the highest was for failed induction (Table 3).

**Table 3:** Indication for emergency cesarean section (*n* = 325)

Indication	Decision to Delivery Time		Total	Median (IQR) min
	Within 30 min	After 30 min		
Cord Prolapse	7	0	7	25(20–26)
APH^a^	6	14	20	37(30–44.5)
NRFHBP^b^	10	68	78	43(35–40)
CPD^c^	9	55	64	40(35–40)
Failed Induction	5	14	19	48(30–63)
Failed VBAC*^4^ and > 1 Scar at Labor	7	32	39	36(34–45)
Breech	5	25	30	42.5(35–56.5)
Grade III Meconium at Latent stage	10	36	46	42.5(34–55.25)
Arrest/Protract disorder with meconium	0	6	6	39(35.75–44.5
Others*^5^	7	9	16	39.5(30–58.75)

^a^*APH* antepartum hemorrhage, ^b^*NRFHBP* nonreassuring fetal heart beat pattern, ^c^*CPD* cephalopelvic disproportion, *^4^*VBAC* vaginal delivery after cesarean delivery, *^5^others (eclampsia, severe preeclampsia, repaired vesico-vaginal fistula at labor, obstructed labor, failed instrument)

### Pre-operative and operative related characteristics

Among the study participants, 23(7.1%) required stabilization before the operation, and 19 (5.8%) were delayed in obtaining operation consent. Material needed for EmCS preparation was available at the labor and delivery ward for 286 (88%) women and the operation Tables (OR table) were busy for nine (2.8%) cases.

### Proportion of recommended decision to delivery interval

**Fig. 1:**
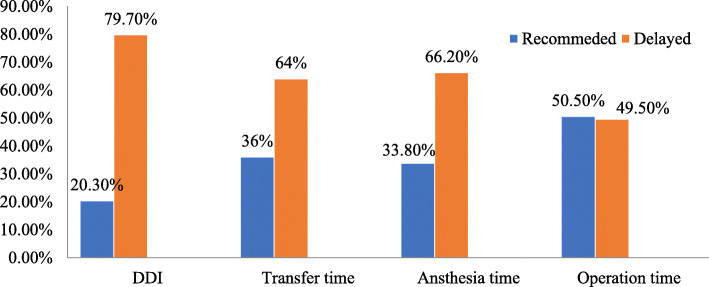
Proportion of decision to delivery and other time interval

The recommended decision to delivery interval was found to be 66 (20.3%) with [95% CI = 15.9–24.7] of EmCS. The median (IQR) time of DDI was 40 (34–50) minutes. Emergency cesarean section was not performed in 44 (13.5%) women until 1 h after the decision time (Fig. 1). The proportion of decision to delivery interval was almost similar in relation to the day of the decision for EmCS, with 20.8% on weekdays and 19% on weekends and public holidays.

### Factor associated with decision to delivery interval

In the bivariable analysis, residence, referral from another institution, transfer time, the status of surgeons, type of anesthesia, operation time, and time of decision were statistically significant at *p*-value< 0.25 level of significance. Residence, referred from other institutions, transfer time, the status of surgeons, type of anesthesia, and time of decision remained significant in the multivariable model. Women who were transferred to the operation theater before 15 min were 5.26 times more likely to have recommended DDI than women who were transferred after 15 min [AOR = 5.26, 95%CI = 2.65–10.46]. In addition, women whose EmCS was performed under general anesthesia were 4 times more likely to have recommended DDIs than women who were performed under regional anesthesia [AOR = 4, 95% CI = 1.6–10]. The findings showed that EmCS performed in the daytime generally had a shorter DDI when compared with the nighttime [AOR = 2.49, 95%CI = 1.26–4.92] (Table 4).

**Table 4:** Bivariate and multivariate analysis of factor affecting decision to delivery time interval (*n* = 325)

Variables	Decision to delivery Interval		COR(95%CI)	AOR(95%CI)
	≤30 min n(%)	> 30 min n(%)		
**Residence**				
Urban	54	174	2.19(1.1–4.33)	
Rural	12	85	1	
**Referred**				
No	27	68	1.94 (1.1–3.41)	2.50(1.26–5.0)*
Yes	39	191	1	
**Transfer Time**				
≤ 15 min	48	69	7.34(3.99–13.48)	5.26(2.65–10.46)**
> 15 min	18	190	1	
**Status of Surgeons**				
Senior	43	88	5.86(2.7–12.68)	2.95(1.3–6.7)*
Resident	9	108	1	
IESO	14	63	2.67(1.09–6.5)	0.67(0.24–1.85)
**Anesthesia type**				
General	14	24	2.63(1.28–5.44)	4(1.6–10)*
Regional	52	235	1	
**Operation time**				
≤ 5 min	38	126	1.43 (1.2–2.5)	
> 5 min	28	133	1	
**Time of Decision**				
Day	46	139	1.98(1.12–3.54)	2.5(1.26–4.92)*
Night	20	120	1	

*p < 0.05, **p ≤ 0.001, IESO (Integrated Emergency Surgical Officers)

## Discussion

This study found that 20.3% of EmCS were performed within the recommended time interval of DDI. This is in line with a study conducted in Gondar, Ethiopia, which was 19.3% [19]. This may be due to the similarity in the accessibility of logistics in hospitals and the practice and experience of professionals. This finding is less than that of studies conducted in Denmark and Oman with recommended DDIs of 87.5 and 60.8%, respectively [7, 20]. The difference may be due to general infrastructure and economic differences in general from those countries. Specifically, in Denmark, a full-scale simulation and color-based multidisciplinary operative room team training was provided over the country to shorten the DDI. In Oman, programs were designed and implemented to shorten DDIs. On the other hand, this finding is greater than that of a study conducted in Nigeria, which was 0.9% of EmCS. This discrepancy may be due to a lack of funds for surgical materials and the absence of post-service billing in the study area of Nigeria, as patients’ relatives usually pay surgical fees before the operation is performed. The findings of this research were also greater than those of studies conducted in Tanzania and Kenya, which were 12 and 3%, respectively [8, 10]. The difference may be that studies in both countries were conducted before the initiation of saving lives through the SaLTS) initiative in East Africa, which was started in late 2016.

In this study, women who were transferred to the operating room before 15 min showed a statistically significant association with recommended DDI than women who were transferred after 15 min. This finding was consistent with study findings in India, Nigeria, Kenya, and Gondar, Ethiopia [9, 10, 19, 21, 22].

This study found that women whose EmCS was performed under general anesthesia were four times more likely to have recommended DDIs than women who were performed under regional anesthesia. The findings of this study were in line with those of studies conducted in Israel, Saudi Arabia, Indonesia, and Norway, which showed that general anesthesia shortened the DDI compared to regional anesthesia [17, 23–25]. The similarity may be a delay in regional anesthesia as a result of technical problems in inducing [9, 22, 24], and stabilization of clients before regional anesthesia is needed [18].

Emergency C/S performed in the daytime generally had a shorter DDI when compared with the night time. This result was comparable to two consecutive studies conducted in Singapore (*p* < 0.05) [21, 26]. However; studies conducted in Norway, Thailand, and Uganda stated that EmCS performed during the daytime had prolonged DDI than those performed at night [23, 27, 28]. The increased number of staff during the daytime for emergency response than night and easy accessibility of logistics and laboratory even from private at day time in our study setting may explain the difference. In the study areas of Thailand and Uganda during daytime operation, the tables were occupied by elective surgery [27, 28].

Women whose EmCS was performed by seniors were two-point nine times more likely to have recommended DDIs than those made by residents. The same result was observed in Norway, with a mean duration difference of 6 min [23]. This could be because seniors were more experienced and had more exposure than residents. A Study in Singapore and Hong Kong showed no significant differences in DDI between senior surgeons and residents. The presence of regular daily drills for residents in both Singapore and Hong Kong study settings may explain this difference [21, 29].

Women who were directly admitted at these hospitals had two-point-five times more likely to have recommended DDI as compared to those who were admitted after referred from other institutions. As the investigators reviewed, no studies were conducted for comparison. The result may be explained referral cases may be more complicated and may need stabilization before the operation.

### Limitations of the study

The study is prone to Hawthorne effects; the health care provider may know they are being observed in a research study their behavior may be influenced by what they assume to be the researcher’s expectations, and observer bias may also affect our outcome of interest. This study does not address the short-and long-term effects of DDI on feto-maternal outcomes.

## Conclusion

Decision-to-delivery interval within the recommended time is not achieved. Being a referral, time of day of EmCS, type of anesthesia, time taken for client preparation and transfer to the operation theater, and the status of surgeons are associated factors of DDI. Hence, to address institutional delays in EmCS, providers and facilities should be prepared in advance and ready for rapid emergency action.

## Supplementary Information

**Additional file 1.**

## Data Availability

The datasets analyzed during the current study are available from the corresponding author upon reasonable request.

## Abbreviations

AOR: Adjusted odds ratio
CI: Confidence interval
CS: Cesarean section
DDI: Decision to delivery interval
EmCS: Emergency cesarean section
