# Supplementary material for: Decision to delivery interval and associated factors for emergency cesarean section: a cross-sectional study
Source: BMC Pregnancy Childbirth. 2021 Mar 20;21:224. doi: 10.1186/s12884-021-03706-8 (PMC7981954; doi:10.1186/s12884-021-03706-8)
Supplement: Supplementary file 1 — Additional file 1. [file 12884_2021_3706_MOESM1_ESM.docx]

**The tool was developed after an extensive review of the literature and modified and finalized according to the suggestions and recommendations of local experts** [8-12, 17-20].

Both the interview administered questionnaires (from Q♯101 up to Q♯304) and observational chick list (from Q♯305 up to end) was attached below.

Annex II : English Version Questionnaire

|  | **Part I: Question on socio-demographic characteristics** | | | |
| --- | --- | --- | --- | --- |
| S.R | Question | Possible answer | Code | Skip |
| 101 | Age in year |  |  |  |
| 102 | Marital Status | Single | 1 |  |
|  |  | Married | 2 |  |
|  |  | Divorced | 3 |  |
|  |  | Widowed | 4 |  |
| 103 | Educational status | Can't read and write | 1 |  |
|  |  | Read and write | 2 |  |
|  |  | Elementary | 3 |  |
|  |  | High school | 4 |  |
|  |  | Preparatory | 5 |  |
|  |  | Diploma and above | 6 |  |
| 104 | Occupation | House wife | 1 |  |
|  |  | Government Employee | 2 |  |
|  |  | Daily labor | 3 |  |
|  |  | Farmer | 4 |  |
|  |  | Merchant | 5 |  |
|  |  | Other(specify)______________ | 6 |  |
| 105 | Place of residence | Urban | 1 |  |
|  |  | Rural | 2 |  |

| **Part II: Obstetrics related questions** | | | | |
| --- | --- | --- | --- | --- |
| 201 | Number of pregnancy |  |  |  |
| 202 | Number of delivery |  |  |  |
| 203 | Did you have ANC follow-up in your recent pregnancy? | Yes | 1 |  |
|  |  | No | 2 |  |
| 204 | How many times did you visited ANC clinic? | One | 1 |  |
|  |  | Two | 2 |  |
|  |  | Three | 3 |  |
|  |  | Four and above | 4 |  |
| 205 | Did you counseled on BPCR plan during ANC follow up. | Yes | 1 |  |
|  |  | No | 2 |  |
|  |  | No | 2 |  |
| 206 | Are you referral | Yes | 1 |  |
|  |  | No | 2 |  |
| 207 | Do you have history of delivery by EmCS( Only for parous women) | Yes | 1 |  |
|  |  | No | 2 |  |

| **Part III: Decision to delivery interval related questions** | | | | | | |  |
| --- | --- | --- | --- | --- | --- | --- | --- |
| S.N | Question | | Possible answer | | Code | Skip |  |
| 301 | Day of week of decision for emergency cesarean section | | Weekday | | 1 |  |  |
|  |  |  | Weekend/public holiday | | 2 |  |  |
| 302 | Time of day of decision for emergency cesarean section | | Day | | 1 |  |  |
|  |  |  | Night | | 2 |  |  |
| 303 | Indication for cesarean section( if there is more than one take the most emergent) | | ––––––––––––––––––––––––––– | |  |  |  |
| 304 | Rank ( status) of surgeon | | Senior | | 1 |  |  |
|  |  |  | Residents |  | 2 |  |  |
|  |  |  | IESO | | 3 |  |  |
|  |  |  | Others(specify) | | 4 |  |  |
| **Fill by Observation** | | | | | | | |
| 305 | | Time at decision for emergency cesarean section |  | |  |  | |
| 306 | | Need to stabilize the patient before operation | Yes | |  |  | |
|  |  |  | No | |  |  | |
| 307 | | Delay in obtaining consent | Yes | |  |  | |
|  |  |  | No | |  |  | |
| 308 | | Presence of material for preparations at labor ward | Yes | |  |  | |
|  |  |  | No | |  |  | |
| 309 | | Time while women arrival at Operation room |  | |  |  | |
| 310 | | Free, functional OR table present | Yes | |  |  | |
|  |  |  | No | |  |  | |
| 311 | | Anesthesia induction time |  | |  |  | |
| 312 | | Type of anesthesia | General anesthesia | | 1 |  | |
|  |  |  | Regional anesthesia | | 2 |  |  |
| 313 | | Incision starting time | _______________ | |  |  | |
| 314 | | Baby out time | _______________ | |  |  | |
| The interviewer should calculate time interval for each and fill accordingly | | | | | | | |
| 315 | | Transfer time |  | |  |  | |
| 316 | | Anesthesia time |  | |  |  | |
| 317 | | Operation time |  | |  |  | |
| 318 | | DDI in minutes |  | |  |  | |

The End!!!

Thank You!!!
